# 4249 Markers of mitochondrial biogenesis, fusion and architecture are disturbed in PBMC from war veterans with posttraumatic stress disorder (PTSD)

**DOI:** 10.1017/cts.2020.307

**Published:** 2020-07-29

**Authors:** Silvana Andric, Aleksandra Markovic, Mirko Milosevic, Sava Radovic, Isidora Starovlah, Jelena Brkljacic, Danijela Vojnovic Milutinovic, Gordana Matic, Tatjana Kostic

**Affiliations:** 1 LaRES/ChronAge, CeRES, Faculty of Sciences, University of Novi Sad; 2 LaRES/ChronAge, Faculty of Sciences, University of Novi Sad; 3 IBISS, University of Belgrade

## Abstract

OBJECTIVES/GOALS: The aim of this study was to define the transcription profiles of the molecular markers of mitochondrial biogenesis and fusion/architecture, and the markers of mtDNA copy numbers in the peripheral blood mononuclear cells (PBMCs) from war veterans with/without post-traumatic stress disorder (PTSD). METHODS/STUDY POPULATION: The peripheral blood mononuclear cells (PBMCs) from war veterans with/without post-traumatic stress disorder (PTSD) were used to monitor transcription profile of the molecular markers of mitochondrial biogenesis and fusion/architecture, as well as the markers of mtDNA copy numbers. The human male immortalized monocytes were exposed *in vitro* to hormonal markers of PTSD in order to monitor the effects of each particular hormonal marker on the molecular markers of mitochondrial biogenesis and fusion/architecture, as well as the markers of mtDNA copy numbers. RQ-PCR analyses were used to define transcriptional profile of above mentioned markers. RESULTS/ANTICIPATED RESULTS: The transcription profiles of above mentioned markers were disturbed, with high individual variability within the groups. A significant increase in the expression of the *PPARGC1A* transcript was observed in a group of subjects with current PTSD, as well as in the subjects with “life-time” PTSD, compared to healthy controls. *PPARGC1B, NRF2 and MFN2* transcripts increased only in PBMCs of “life-time"-PTSD, while the level of transcripts for other investigated genes and the ratio of markers of mtDNA copy numbers showed no significant difference between groups. The *in vitro* results showed parallelism with the results obtained using the PBMCs from the subjects of the PTSD study. DISCUSSION/SIGNIFICANCE OF IMPACT: Although preliminary (the analysis require a larger number of subjects), the results are first findings and a solid base for further extensive multidisciplinary research in order to clarify the molecular mechanisms for the prevention and treatment of trauma-induced pathological conditions.

